# Clinical, immunological, and genetic landscape of common variable immunodeficiency in Morocco: a nationwide multicenter study

**DOI:** 10.3389/fimmu.2025.1602820

**Published:** 2025-07-09

**Authors:** Abire Allaoui, Abderrahmane Moundir, Ibtihal Benhsaien, Fatima Ailal, Jalila El Bakkouri, Khadija Echchilali, Abdelhamid Naitlho, Hassan El Kabli, Ahmed Aziz Bousfiha, Mina Moudatir

**Affiliations:** ^1^ Clinical Immunology, Inflammation and Allergy Laboratory (LICIA), Faculty of Medicine and Pharmacy, University of Hassan II Casablanca, Casablanca, Morocco; ^2^ Immunopathology-Immunotherapy-Immunomonitoring Laboratory, Faculty of Medicine, Mohammed VI University of Health and Sciences, Casablanca, Morocco

**Keywords:** common variable immunodeficiency, inborn errors of immunity, genetic variants, immune dysregulation, Morocco, Africa, next-generation sequencing

## Abstract

**Background:**

Common Variable Immunodeficiency (CVID) is the most prevalent symptomatic inborn errors of immunity (IEI), characterized by impaired antibody production, recurrent infections, and immune dysregulation. While extensively studied in Western populations, data from North Africa remains scarce. This study provides the first comprehensive evaluation of the clinical, immunological, and genetic landscape of CVID in a Moroccan nationwide cohort.

**Methods:**

A multicenter, cross-sectional study was conducted across eight university hospitals in Morocco from 2019 to 2025. Sixty-one CVID patients were enrolled according to ESID (European society of immunodeficiency) and MENA (Middle-East and North Africa) guidelines. Clinical, immunological, and genetic data were analyzed. Whole-blood samples were processed for immunophenotyping, and a subset of patients underwent next-generation sequencing (NGS) targeting 474 inborn error of immunity (IEI)-associated genes.

**Results:**

The mean age at diagnosis was 25.9 (SD 18.7) years old, with a diagnostic delay of 6.91 (SD 8.82) years. The most frequent infectious complications were pulmonary infections (88.5%) and gastrointestinal infections (63.9%). Non-infectious complications were present in 49.2% of patients, with predominant features including lymphoproliferation (50.8%), autoimmune cytopenias (39.3%), and granulomatous disease (18%). Bronchiectasis was the most common pulmonary finding (44.3%). Genetic testing (n=25) revealed 19 pathogenic variants in 13 genes, including 14 novel variants, particularly *LRBA, CTLA4, PIK3CD, NFKB1, VAV1*, and *TCF3*. The phenotype-genotype correlation, based on clinical presentation, gene function, and multidisciplinary assessment, was strong in 52.6% of cases.

**Conclusion:**

This study provides Morocco’s first clinical and genetic landscape of CVID, highlighting a high prevalence of consanguinity-associated monogenic defects and a significant burden of infectious and immune dysregulatory complications. Our findings emphasize the need for early diagnosis, multidisciplinary management, and access to targeted therapies in non-Western settings. Further studies with functional validation of genetic variants are warranted to refine precision medicine approaches in CVID.

## Introduction

Common Variable Immunodeficiency (CVID) is the most prevalent symptomatic inborn errors of immunity (IEI) (1), with a prevalence of approximately 1:50,000 to 1:25,000 (2). CVID is characterized by impaired antibody production, recurrent infections, and a broad spectrum of immune dysregulation, including autoimmunity, lymphoproliferation, and malignancies (2–4). Autoimmune cytopenias and lymphoproliferative disorders remain prominent, reinforcing the intricate interplay between immune deficiency and immune dysregulation (2, 5, 6). Despite the availability of immunoglobulin replacement therapy (IgRT) and targeted biologics, challenges persist in optimizing therapeutic strategies, particularly in resource-limited settings (7).

The global landscape of CVID has evolved with advances in immunogenetics and next-generation sequencing (NGS), which have revealed monogenic and polygenic contributions to disease pathogenesis (8–10). Studies from international cohorts have highlighted the complexity of genetic determinants and their heterogeneous correlation with clinical phenotypes (11, 12).

Additionally, the role of environmental factors and regional infectious disease burdens has become increasingly recognized in shaping disease expression and outcomes (5, 13, 14). This clinical and genetic heterogeneity requires a multidisciplinary approach for diagnosis and management.

While extensively studied in Western populations, data from North Africa, particularly Morocco, remain scarce (15–17). This study provides the first comprehensive evaluation of the clinical, immunological, and genetic landscape of CVID in a Moroccan nationwide cohort, comparing our findings with international data. By participating in elucidating the disease spectrum in a non-Western population, our research aims to bridge knowledge gaps, identify diagnostic and therapeutic challenges in non-Western countries, and contribute to the evolving understanding of CVID pathophysiology.

## Methods

A multicenter, cross-sectional, nationwide study of patients diagnosed with CVID was conducted in Morocco from January 2019 to December 2025. Eight university hospitals treating PIDs have participated in the study. The Moroccan-CVID Registry was created under the aegis of the Working Group of the Moroccan Society of Primary Immunodeficiency (MSPID). All patients aged 4 years and above with CVID diagnosis according to the European Society for Immunodeficiencies (ESID) registry working definitions (18) and the Middle East and North Africa Diagnosis Guidelines for Inborn Errors of Immunity (19), with at least decreased levels of two serum immunoglobulins (low IgG and low IgA and/or low IgM, >2 standard deviations below the mean for age) and impaired antibody response or low B memory switched-class lymphocytes, with the exclusion of defined causes of hypogammaglobulinemia such as malignancies, medications, protein loss, or bone marrow failure, were considered eligible.

### Data collection and variables

Data was extracted from the Moroccan-CVID Registry, which systematically compiles sociodemographic, clinical, biological, and immunological data, accompanying co-morbidities, family history, genetic data, treatments, and patient clinical courses. All data were collected by reviewing electronic medical records and included in a predesigned database on SPSS (Statistical Package for the Social Sciences) software.

Demographic data included sex, age at diagnosis, age at clinical onset, diagnostic delay, first PID- related clinical complication (infection, dysimmunity, malignancy, or other), and follow-up time.

Genetic testing, family history, and consanguinity were recorded.

Blood samples were obtained and placed in ethylenediaminetetraacetic acid (EDTA) tubes for complete blood count analysis (white blood cell count, lymphocyte count, and neutrophil count). All patients underwent HIV tests to rule out HIV infection. Fluorescently labeled monoclonal antibodies specific for T cell markers (CD3, CD4, CD8), B cell markers (CD19, CD20), and NK cell markers (CD16, CD56) were added to the samples and incubated. The enumeration was performed using flow cytometry with a FACSCanto II flow cytometer (from BD Biosciences, San Jose, CA) and analyzed using FACSDiva software. Serum immunoglobulin (Ig) levels were determined by nephelometry (BNProspec, Siemens). All patients immunoglobulin and lymphocyte subset levels were tested before the first Ig replacement therapy. Patients with transient hypogammaglobulinemia or selective IgA deficiency (normal IgG/IgM) were excluded via repeat testing.

All other diagnostic tests, such as chest X-ray, computer tomography of the chest, endoscopic interventions, sputum/blood/stool cultures, and autoimmunity tests, were evaluated according to each patients clinical course as medically required. Infectious, hematologic and malignant causes were excluded for cases of splenomegaly and lymphadenopathies.

All patients underwent Ig testing and lymphocyte subtype enumeration, but only 36 patients had B lymphocyte subset enumeration, only seven patients had a response to vaccine testing, and only 25 patients had genetic testing due to insufficient facilities and resources.

The immunological phenotype of each patient was studied and classified according to EUROClass (20) classifications.

Genetic, flow cytometry, and immunologic testing were carried out by the Immunology Laboratory, Ibn Rochd University Hospital, and Laboratoire National de Recherche Mohammed VI (LNRM6) and by a deep sequencing array (using a custom-designed Illumina SNP array) with the 474 genes involved in inborn errors of immunity (21).

Variants were sorted to find non-synonymous variants with a minor allele frequency (MAF) of less than 0.01 in the Genome Aggregation Database (gnomAD v3). This helped find variants that might be causal. Our analysis concentrated on IEI genes as categorized by the International Union of Immunological Societies (IUIS) (22). The mutations have to align with the disease’s mode of transmission: one variant for autosomal dominant or X-linked transmission in males and two variants for autosomal recessive transmission. For diseases that can be inherited in a dominant or recessive transmission, the presence of one copy of the mutated gene (heterozygous variant) was deemed adequate for analysis. To prioritize variations with a detrimental effect, in silico pathogenicity predictions were utilized, including the CADD (Combined Annotation Dependent Depletion) approach (23), the SIFT (Sorting Intolerant from Tolerant) method (24), the Polyphen2 (Polymorphism Phenotyping v2) method (25), and the Alpha Missense method (26). Candidate variants were subsequently analyzed for genotype-phenotype correlation. “Strong correlation” referred to a match between the clinical presentation and the known function of the gene based on current literature and in silico predictions, further supported by multidisciplinary team review. Defined as High (≥2 features), Moderate (1 feature), or Low (no known associations). Disease databases, including OMIM and ClinVar, were utilized to assess the genetic variant’s contribution to the disease. All discovered variants were assessed for their potential pathogenicity following the recommendations set forth by the American College of Medical Genetics and Genomics (ACMG) (27). Consequently, variants were categorized as pathogenic, likely pathogenic, of uncertain significance (VUS), likely benign, and benign. Popviz (28) was used to determine the mutation significance cutoff (MSC) with a confidence level of 95%. The significance of each mutation was evaluated during multidisciplinary meetings involving physicians, geneticists, immunologists, and other specialists, informed by clinical status and literature findings.

The follow-up of our patients adheres to a locally applied monitoring protocol: abdominal ultrasound is performed every 6 to 12 months; chest CT scans are repeated every 2 years; pulmonary function tests are conducted annually; and upper and lower gastrointestinal endoscopies are scheduled every 2 years, or earlier if clinically indicated.

### Statistics

To ensure efficient data management, we used a computerized database using SPSS (Statistical Package for the Social Sciences) version 25 software. Using this database facilitated the entry of each patient’s information and simplified the subsequent analyses. The data were subjected to descriptive and statistical analyses. We considered the p-value <0.05 as significant. Qualitative characteristics were presented as percentages, while quantitative characteristics were expressed as mean or median with standard deviation, using the tools available in SPSS software.

### Ethical statement

The patients or parents/guardians of the young participants gave written informed consent to participate in the study and genetic testing. The study was approved by the Ethics Committee of the Ibn Rochd University Hospital in Casablanca.

## Results

### Demographics

**Figure 1:**
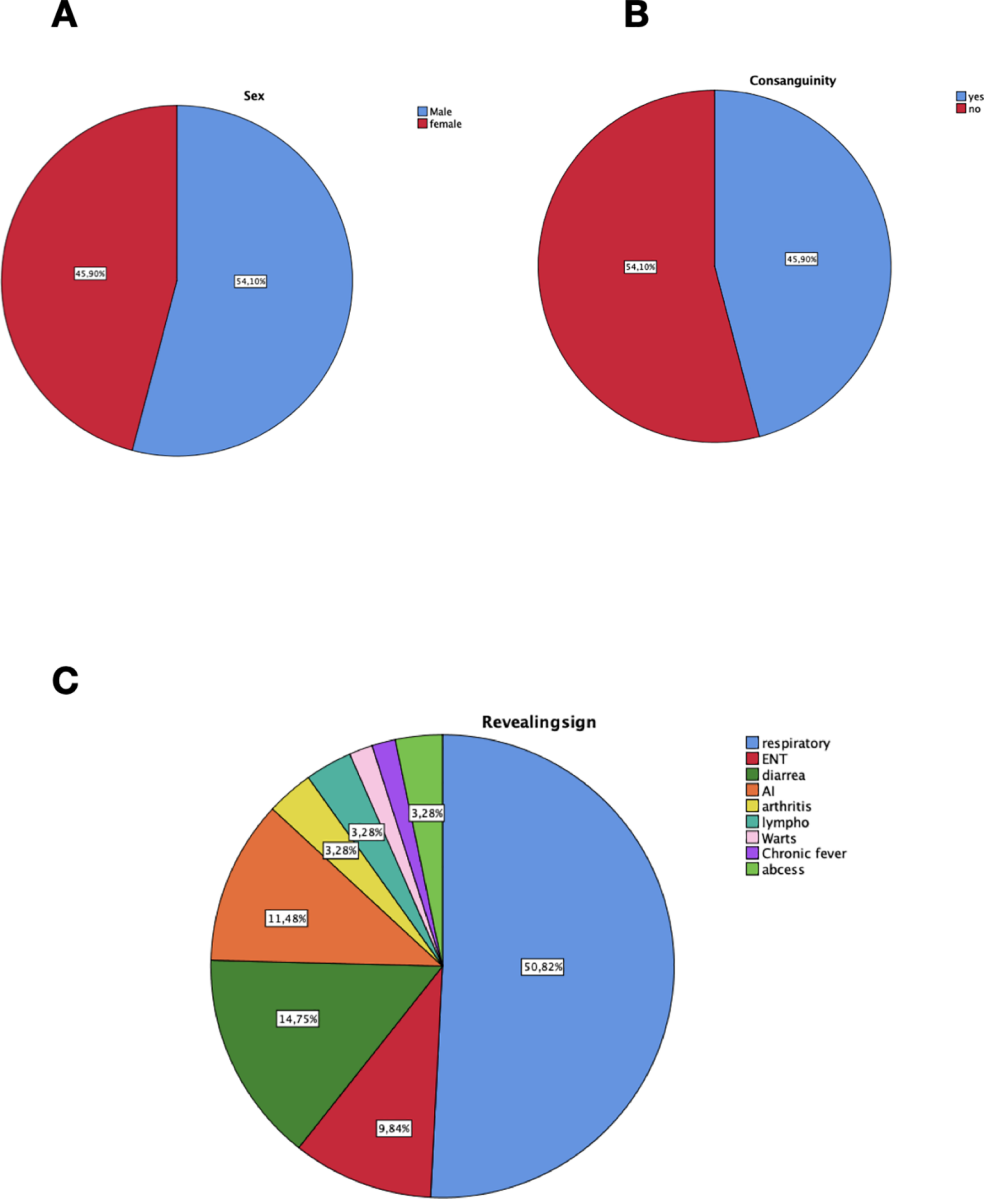
Demographic characteristics and major revealing signs of 61 Moroccan patients with CVID. **(A)** Gender distribution in our cohort. **(B)** Consanguinity distribution in our population. **(C)** Distribution of revealing signs at diagnosis. respiratory, respiratory infections; ENT, Ear nose throat infections; AI, Autoimmunity; lympho, lymphoproliferation, abcess; deep-seated infections.

A total of 61 patients diagnosed with CVID were included. In our cohort, the mean age at diagnosis was 25.9 (SD 18.7) years old, and 33 patients (54.1%) were male (**Figure 1A**). However, the mean diagnostic delay was 6.91 (SD 8.82) years, while the global mean age at characteristic symptom onset was 19.00 (SD 17.28) years old. Most patients initially presented with infectious complications in 80.3% of the cases (n=49), followed by non-infectious complications (19.7%, n=12) (**Figure 1B**). The consanguinity rate was 54.1% (n=33) (**Figure 1C**). A family history of immunodeficiency was noted in one case.

### Infectious complications

Pulmonary infections were the major cause of recurrent infections in 88.5% (n=54), followed by digestive tract infections in 63.9% (n=39), then ENT infections in 50.8% (n=31), and pyelonephritis was noted in one case. Skin, soft tissue, or musculoskeletal infections were noted, respectively, in 3.3% (n=2), 3.3% (n=2), and 1.6% (n=1). Almost 63.9% (n=39) had suffered at least one episode of a major bacterial infection complicated with sepsis in 8.2% (n=5), and pneumonia accounted for most episodes, 60.7% (n=37). Bacterial infections were mostly caused by streptococcus pneumonia, staphylococcus aureus, pseudomonas, salmonella, and Klebsiella pneumonia.

Gastroenteritis was caused by giardia enteritis, norovirus, and enterovirus. Helicobacter pylori was a predominant cause of gastritis. Seven cases (11.5%) of mycobacterium tuberculosis were noted (5 pulmonary infections, 2 adenitis), and two cases of herpes zoster virus infections.

### Non-infectious complications

Non-infectious complications were present in 30 patients (49.2%). The most common immune dysregulation complication was lymphoproliferation with splenomegaly (50.8%, n=31), closely followed by lymphadenopathies and immune cytopenias, each one in 39.3% (n=24), autoimmune hemolytic anemia (AIHA) 11.5% (n=7), immune thrombopenia (ITP) 27.9% (n=17), Evans syndrome (AIHA+ ITP) in 8.2% (n=5), and neutropenia 9.8% (n=6), non-infectious enteropathy in 14.8% (n=9), uveitis, and inflammatory arthritis, each one in 6.6% (n=4). Granulomatosis (sarcoidosis-like disease) was noticed and confirmed histologically in 18% (n = 11) (pulmonary, hepatic, and splenic involvement). Systemic diseases were present in three patients (systemic lupus, rheumatoid arthritis, and psoriatic rheumatism). Autoimmune and inflammatory complications are regrouped in **Table 1**.

**Table 1:** Autoimmune complications in Moroccan patients with CVID.

Autoimmune complications	Frequency	Percentage (%)
ITP	17	27.9
AIHA	7	11.5
Granulomatosis	11	18.0
Vitiligo	5	8.2
Uveitis	4	6.6
Arthritis	4	6.6
Systemic lupus	1	1.6
Rheumatoid arthritis	1	1.6
Psoriatic Rheumatism	1	1.6
Bullous dermatosis	1	1.6

ITP, Immune thrombocytopenic purpura, AIHA, Autoimmune hemolytic anemia.

**Figure 2:**
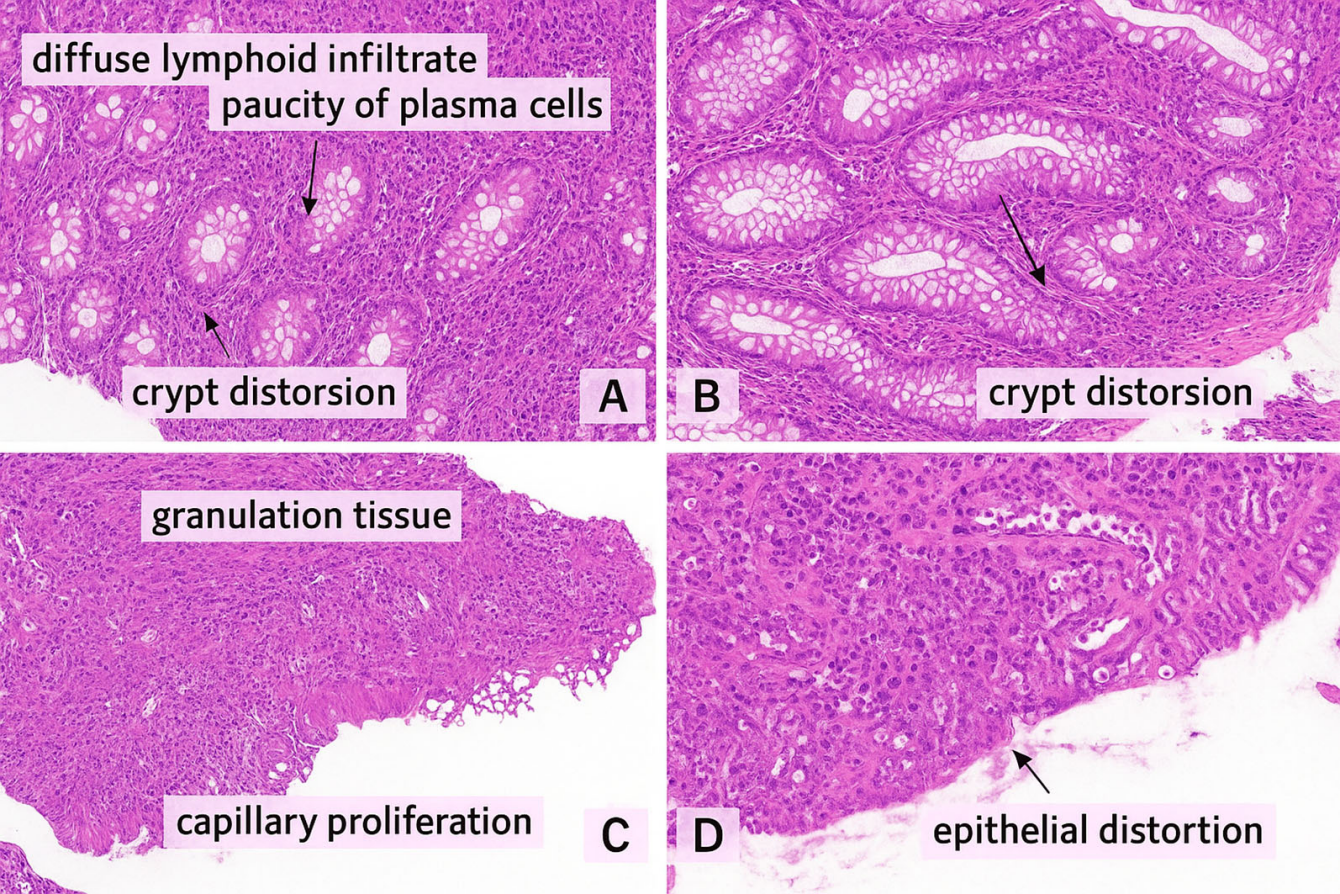
**(A, B)** Colonic mucosa with a dense, diffuse lymphoid infiltrate within the lamina propria, composed predominantly of small lymphocytes, with a striking paucity of plasma cells. No epithelioid granulomas or giant cells were observed (Hematoxylin & Eosin stain) **(C, D)** Colonic biopsy showing granulation tissue characterized by a mixed inflammatory infiltrate, fibrino-leukocytic debris, and capillary proliferation. The colonic epithelium exhibits glandular dedifferentiation and reduced mucin production (Hematoxylin & Eosin stain). These microscopic findings are consistent with Crohn-like enterocolitis in a 26 years old female Moroccan patient with *TNFRSF13B* mutation associated to *CD21* deficiency.

Coeliac-like and Biermer-like diseases were noted in 14.8% (n=9) and 3.3% (n=2), respectively. IBD-like disease (inflammatory bowel disease) was found and confirmed histologically in 13.1% (n=8) (**Figure 2**). Liver disease was represented by hepatitis, hepatomegaly, and liver nodules, found in 4.9% (n=3) and 3.3% (n=2), respectively, complicated with portal hypertension in one case.

**Figure 3:**
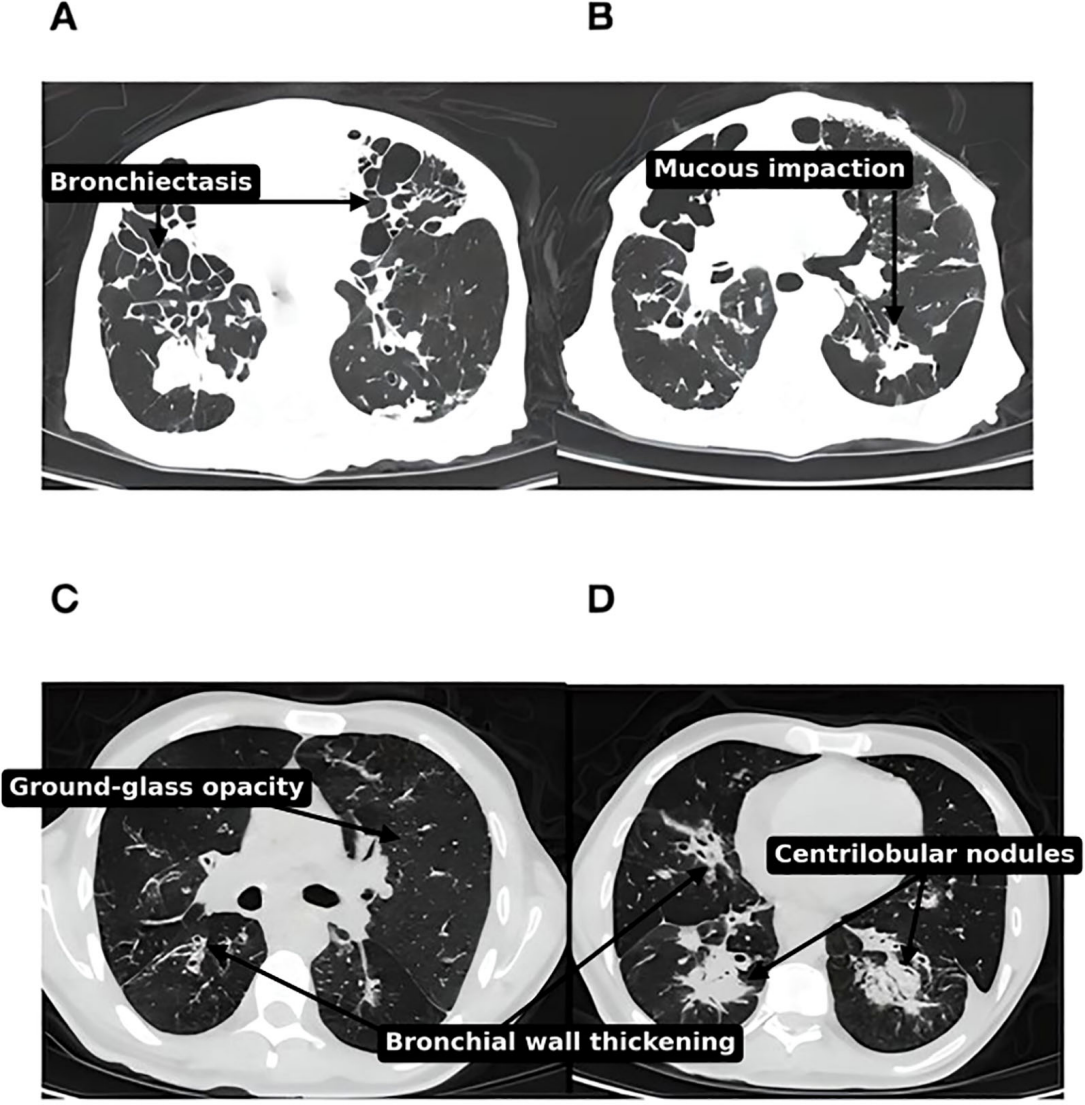
**(A, B)** Thoracic CT scan, parenchymal window, bilateral diffuse nodules and diffuse cystic bronchiectasis in a 78 years old Moroccan patient with CVID (no gene was identified). **(C, D)** Thoracic CT scan, parenchymal window, of diffuse nodules, reticulations and ground-glass opacities. Apart from CVID-ILD features, there are also diffuse bronchiectasis with mucoid impaction pointing to surinfection, in 55 years old male Moroccan patient with *TNFRSF13B* mutation.

Dyspnea and cough were the most common pulmonary symptoms. High-resolution computed tomography (HRCT) was performed in 54 patients (88.5%) (**Figures 3**). Bronchiectasis was the most common imaging finding (44.3%, n=27). Lung parenchymal involvement was observed in 11.5% (n=7) of patients, and the most commonly described radiological pattern was compatible with lymphocytic interstitial pneumonia in 8.2% (n=5). Combined lymphocytic interstitial pneumonia and granulomatosis (GLILD) was not confirmed histologically in any of our patients. Three patients developed chronic respiratory failure requiring oxygen therapy.

**Figure 4:**
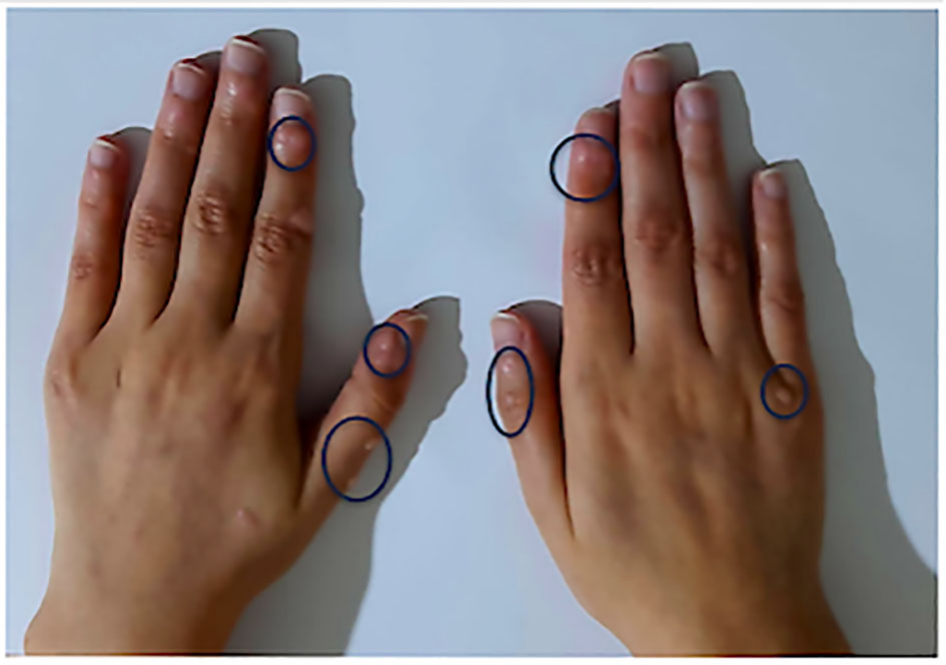
Warts in a patient with LRBA deficiency from our cohort. Warts are circled in blue.

Skin lesions were reported in 14 patients in our cohort (22.9%). Vitiligo was the main skin lesion in 8.2% (n=5), warts in 6.6% (n=4) (**Figure 4**), poliosis, bullous dermatosis, and psoriasis in one case each. Atopic dermatitis and drug allergy were observed in two patients (3.3%).

Neurological immunodemyelinating lesions were found in one patient who was admitted to intensive care with impaired consciousness and died during this hospitalization following a septic choc.

Three patients presented with cancers in their clinical course: one patient with Burkitt lymphoma, one patient with marginal-zone lymphoma (gastric MALT lymphoma), and one patient with colorectal cancer.

### Immunological features

There was significant variability in immunoglobulin levels and the numbers of B and T cell subpopulations between patients. Immunoglobulin levels upon diagnosis and lymphocyte subpopulation cell counts are presented in **Table 2**.

**Table 2:** Immunoglobulin levels and lymphocyte subpopulations count in the CVID Moroccan cohort.

Immunoglobulin levels and lymphocyte subpopulations count	N	Minimum	Maximum	Mean	Standard deviation
IgM	61	02	4,05	7465	79215
IgG	61	00	8,95	3,6920	2,36681
IgA	61	00	4,47	8196	1,27208
CD3	61	76,90	3663,00	1609,7213	943,02796
CD4	61	27,40	1944,00	701,6705	466,92413
CD8	61	36,30	2640,00	803,1131	647,85247
CD19	61	,00	2243,00	221,4233	354,13861
CD16CD56	61	,05	783,00	168,3308	167,61632

N, Number; IgM, immunoglobulin M; IgG, immunoglobulin G; IgA, immunoglobulin A; CD, The cluster of differentiation.

### Patient management and outcomes

Fifty-three patients (86.9%) were under active treatment with immunoglobulin replacement treatment (IgRT). All of the treated patients were on the intravenous route (IVIgRT).The prescribed dose was 400 mg/kg/month (ranging from 300–600 mg/kg). Good outcome with a reduced incidence of recurrent and severe infections and subsequent hospitalizations was noted in 65,6% (n=40) of the total number of patients, this percentage increases to 90% (n=36/40) among the patients who received immunoglobulines.

75.4% (n=46) of the patients were given antibiotics as a preventative measure. Prophylactic trimethoprim- sulfamethoxazole (TMP-SMX) 160/800 mg three times a week was initiated in patients with recurrent respiratory infections, independent of CD4+ counts. Patients with bronchiectasis were given azithromycin 500 mg three times a week.

Thirty patients (49.2%) received a prescription for corticosteroids. Ten patients (16.4%) were given immunosuppressants or immunomodulators to help with autoimmune and inflammatory complications of CVID, including cytopenias, granulomatosis, lymphoproliferation, and autoimmune disorders. Azathioprine had been administered to three patients. Two patients were on mycophenolate mofetil. Methotrexate was used in two patients, and one patient was put on hydroxychloroquine.

Five patients (8.2%) also used targeted therapies. Rituximab therapy was administered in three cases, one to manage immune cytopenia and the others to complete the chemotherapy regimen for Burkitt lymphoma and marginal-zone lymphoma. Two patients with *LRBA* and *CTLA4* deficiency were put on abatacept then infliximab to manage rheumatological and intestinal inflammation. No patient benefited from bone marrow transplantation.

Three patients have died, meaning a mortality rate of 4.9% in our cohort at the time of data collection, with a mean age at death of 50 years old (SD 19.8). Dysimmune complications (severe autoimmune enteropathy) and AA amyloidosis in a patient with *TCF3* mutation and septic choc in two patients without genetic confirmation, were the causes of death.

### Genetics

Twenty-five patients were tested for genetics (41%). Following an in silico analysis using predictive methods (CADD, SIFT, Polyphen2), we identified 19 putative pathogenic variants in 13 genes, of which 14 (73.7%) are novel and 5 (26.3%) have been previously documented. Biallelic variants were present in 6 patients, which corresponds to 31.6% of patients with a potential genetic diagnosis. X-linked variants were present in one patient (5.26%), and monoallelic variants were present in 12 patients (63.15%). Based on ACMG guidelines, 12 (63.15%) of the variants found were labeled as VUS, 6 (31.5%) as pathogenic, and 1 (5.26%) as likely pathogenic. At the molecular level, there were 12 missense (63.15%), 3 splice donors (15.8%), 2 frameshifts (10.5%), 1 codon stop (5.26%), and 1 deletion (5.26%). All details are in **Table 3**.

**Table 3:** Potential causal variant classification and in silico analysis.

Gene variant (Transcript ID)	Zygosity	Heredity	Previously reported(*)	Previously reported(*)	ACMG variant class	Molecular consequences	CADD score	SIFT	Polyphen	AlphaMissense
*CTPS1:c.262C>G (p.Leu88Val)* (ENSG00000171793)	Heterozygous	AR	No	High	VUS	Missense	23.0	Deleterious (0.02)	Benign	0.1228
*VAV1:c.2411A>C (p.Lys804Thr)* (ENSG00000141968)	Heterozygous	AD	No	High	VUS	Missense	23.1	Deleterious (0.04)	Potentially damaging	0.34
*PIK3CD:c.2353C>T (*p.Arg785Trp) (ENSG00000171608)	Homozygous	AD	No	Moderate	VUS	Missense	34	Deleterious	Potentially damaging	0.93
CTLA4: c.216dup(p.Val73Serfs*6) (ENSG00000163599)	Heterozygous	AR	No	High	pathogenic	Frameshift	34	Deleterious	Pathogenic	:
NFKB1:c.877_902delinsTTTTTTTTC (p.Gly293Phefs*10) (ENSG00000109320)	Heterozygous	AD	No	Moderate	pathogenic	Missense	34	Deleterious	Pathogenic	:
*STK4: c.1305 + 1G>A* (ENSG00000101109)	Homozygous	AR	Yes (PMCID: PMC11437448)	Moderate	VUS	Splice donor	34	Deleterious	Potentially damaging	:
*LRBA:c.6505G>A(p.Asp2169Asn)* (ENSG00000198589)	Heterozygous	AD	No	Moderate	VUS	Missense	28.9	Deleterious (0.02)	Potentially damaging	–
LRBA:c.5060_5067del (p.Asn1687Serfs*21) (ENSG00000198589)	Homozygous	AR	No	High	pathogenic	Frameshift	35	::	:_	:
*STK4*:*c.750G>A* (p.W250*) (ENSG00000101109)	Homozygous	AR	Yes (PMCID: PMC11437448)	Moderate	VUS	Stop codon	42	Deleterious	Potentially damaging	–
*TNFRSF13B*: c.604C>T (p.Arg202Cys) (ENSG00000240505)	Heterozygous	AR	No	High	VUS	Missense	5.3	Tolerated	Benign	0.06
*CR2:c.2460G*>T(p.Gln820His) (ENST00000342505.5)	Heterozygous	AR	No	High	VUS	Missense	14.5	Tolerated	Potentially damaging	–
*TCF3*, Exon 2, c.16A>G (p.Arg6Gly (ENSG00000071564)	Heterozygous	AR or AD	No	High	VUS	Missense	24.4	Deleterious	Potentially damaging	0.25
*LRBA*:c.476_549 + 580del (ENSG00000198589)	Homozygous	AR	No	High	Potentially pathogen	Splice donor	:_	:_	:_	:
*CD19:c.1260C>A*(p.Phe420Leu) (ENSG00000177455)	Heterozygous	AR	No	Moderate	VUS	Missense	16.70	Tolerated	Potentially damaging	0.71(pathogenic)
*SH3KBP1 c.662A>G (p.Asp221Gly)* (ENSG00000147010)	Hemizygote	XL	No	Moderate	VUS	Missense	26.6	Deleterious	Potentially damaging	0.71(pathogenic
*TNFRSF13B c.260T>A (p.Ile87Asn)* (ENSG00000240505)	Heterozygous	AR	Yes (PMCID: PMC4829724)	High	pathogenic	Missense	24	Deleterious	Potentially damaging	0.71(pathogenic
*RNASEH2B c.529G>A (p.Ala177Thr)* (ENSG00000136104)	Heterozygous	AR	Yes (PMCID: PMC4544753)	Low	pathogenic	Missense	20.6	Deleterious	Potentially damaging	0.11
*LRBA, Deletion (Exons 30-34)* *(*ENSG00000198589)	Homozygous	AR	Yes (PMCID: PMC3370280)	High	pathogenic	Deletion	:_	::	:_	:
*EPG5, Exon 33, c.5680A>G (p.Ile1894Val) (*ENSG00000152223)	Heterozygote	AR	No	Low	SUV	Missense	16.42	Tolerated	Benign	78

*Previously reported in Clinvar and OMIM as related to a clinical condition. AD, autosomal dominant; AR, autosomal recessive; XL, X-linked; Low correlation: No clear features associated with the genetic defect. Moderate correlation: One known feature is associated with the genetic defect. High correlation: Two or more known features are associated with the genetic defect. For variant analysis, we used *in silico* prediction tools: SIFT, PolyPhen, CADD score, and Alpha Missense.

**Figure 5:**
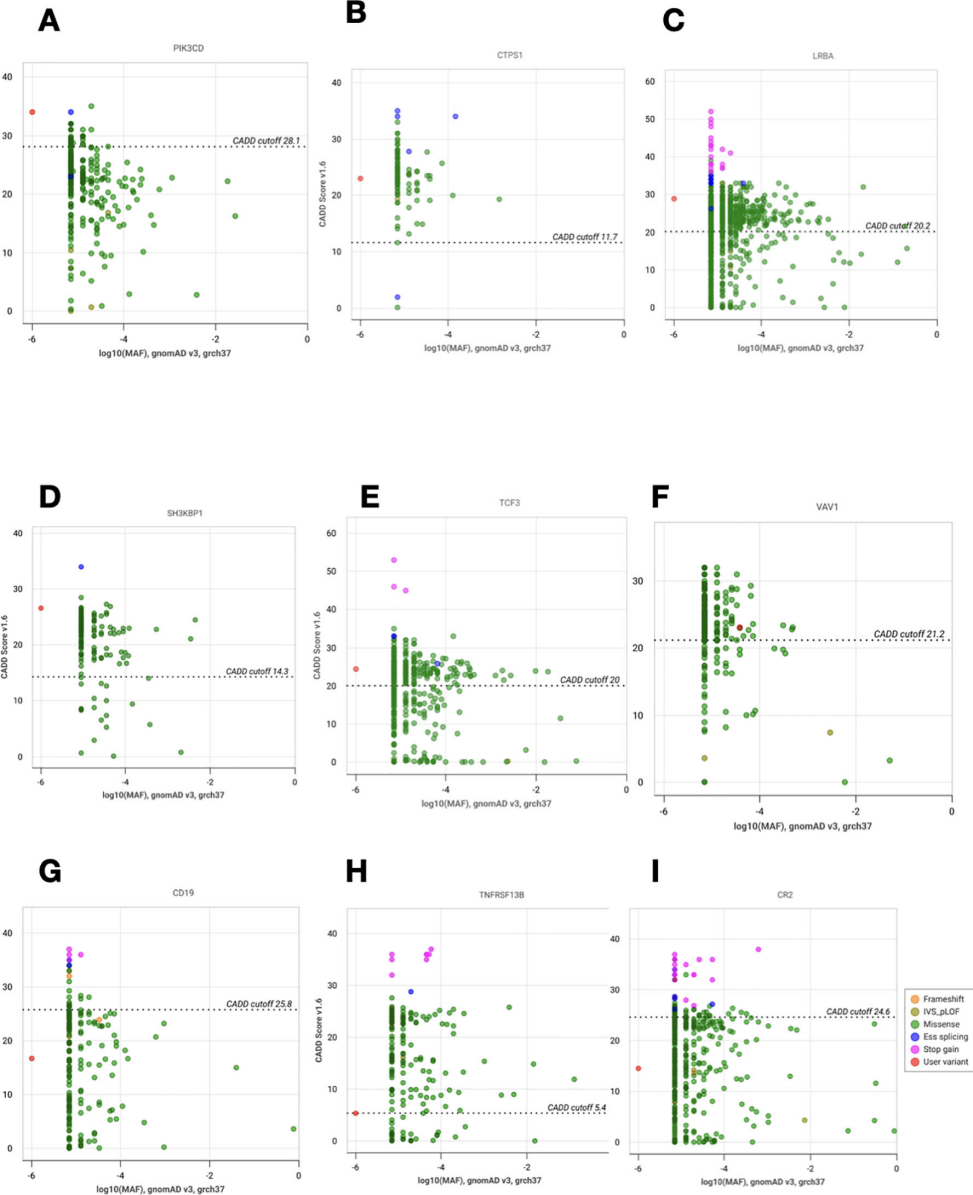
CADD *vs*. MAF plot of novel variants with high or moderate phenotype- genotype correlations. **(A)** Analysis of *PIK3CD* (p.Arg785Trp). **(B)** Analysis of *CTPS1* (p.Leu88Val). **(C)** Analysis of *LRBA* (p.Asp2169Asn). **(D)** Analysis of *SH3KBP1* (p.Asp221Gly). **(E)** Analysis of *TCF3* (p.Arg6Gly). **(F)** Analysis of *VAV1* (p.Lys804Thr). **(G)** Analysis of *CD19* (p.Phe420Leu). **(H)** Analysis of *TNFRSF13B* (p.Arg202Cys). **(I)** Analysis of *CR2* (p.Gln820His). The vertical and horizontal axes show combined annotation- dependent depletion scores (CADD) and minor allele frequencies (MAF), respectively. The CADD cutoff represents the MSC (mutation significance cutoff) on CADD with CI:95%. The red dot corresponds to the new variant and the other dots to the other variants reported for each gene.

We looked for all characteristics linked to the variant or gene to determine how each genotype affected the clinical phenotype. Phenotype-genotype correlation was high in 10 (52.6%) patients, moderate in 7 (36.84%), and low in 2 (10.52%). CADD *vs*. MAF plots showing new potential variants with high or moderate phenotype-genotype correlations are in represented in **Figure 5**. The identified potential causal variants were associated with CVID related-genes in 3 patients: *CD19*, *TNFRSF13B(TACI)*, the association between *TNRFSF13B (TACI)* and *CR2* (CD21 deficiency) in one patient. The variants were associated with CVID-like diseases in ten patients: *LRBA* in 4 cases, *CTLA4* (1 case), *PIK3CD* (1 case), *NFKB1* (1 case), *VAV1* (1 case), *TCF3* (1 case), *SH3KBP1* (1 case). Variants associated with other immune dysregulation defects were present in six patients: *STK4* deficiency in 3 cases, *RNASEH2B* (1 case), *CTPS1* (1 case), and *EPG5* (1 case). All the patients with potential causal variants are detailed in **Table 4** with their clinical phenotypes, immunological features, therapeutics, and outcomes.

**Table 4:** Potential genetic variants identified in the Moroccan cohort and their clinical and immunological characteristics, and outcomes.

Patient identifiers	Gene variant	Other variants	Gender	Age* (onset of illness)	Age **at diagnosis	Clinical features	Particular features	Immunological features	Euroclass	Therapeutic implications: Immunosuppressants	Therapeutic implications: Targeted therapy	Outcome
***P1***	***VAV1***	MEFV/LIG4	F	4	16	Recurrent infections, vitiligo, Biermer and IBD-like conditions	daughter with FMF Angiodeme	Normal CD3, CD4,CD8CD19, CD16 Low	**SmB-CD21low**	No	No	Alive
***P3***	***LRBA***		F	8	15	Lymphoproliferation, autoimmune cytopenias, IBD-like, repeated infections	Uveitis	Normal CD3, CD4,CD8 CD16, Low CD19	**B absent**	Mycophenolate mofetil+ hydroxychloroquine	Abatacept then infliximab	Alive
***P14***	***CTPS1***		M	21	29	Lymphoproliferation, autoimmune cytopenias, repeated infections	Burkitt lymphoma, EBV viremia	Normal CD3, CD4,CD8CD19 CD16	**SmB-TrNorm**	No	No	Alive
***P16***	***PIK3CD***		M	5	9	Repeated infections, autoimmune cytopenias	Heart disease, Hyperpigmented skin spots	Normal CD3, CD4,CD8CD16 Low CD19	**B absent**	No	No	Alive
***P60***	***NFKB1***	CR2 VUS (CD21 deficiency)	M	2	6	Respiratory infections, lymphoproliferation, autoimmune cytopenias	—	ALPS profile (increased double negatives, high vitamin B12, normal IL 10)	:::	No	No	Dead
***P27***	***LRBA***	Caspas 10	M	4	65	Repeated infections,Sarcoidosis-like	colon cancer	Normal CD3, CD4,CD8CD19 CD16	**SmB-TrHi**	No	No	Alive
***P28***	***TCF3***		M	13	20	Repeated infections, coeliac-like, lymphoproliferation	Uveitis AA amylose	Normal CD3, CD4,CD8CD19 CD16	**SmB-CD21low**	No	No	Dead
***P33***	***CTLA4***		M	13	20	Lymphoproliferation, recurrent septic arthritis, IBD-like, autoimmunity	Psoriatic arthritis	Normal CD3, CD4,CD8 Low CD19 CD16	**B absent**	Methotrexate	Abatacept	Alive
***P34***	***CD19***	NOD2(BLAU)/PRKDC	F	20	33	Repeated infections,Lymphoproliferation, Sarcoidosis-like,autoimmune cytopenia	Hepatitis, Shingles	Normal CD3, CD4,CD8CD19 CD16	**SmB-TrNorm**	No	No	Alive
***P40***	***SH3KBP1***	HYOU1/IFNAR2	M	10	12	Repeated infections	Eosinophilia, Recurrent meningitis (normal CH50,C3,C4)	Normal CD3, CD4,CD8CD19 CD16	**SmB-TrNorm**	No	No	Alive
***P47***	***STK4***	Caspas 10/IRF2BP2 /TCF3	M	10	12	Repeated diarrhea episodes, warts, pityriasis versicolor-like skin lesions	Affected sister HPV-related skin disease,	Low CD19 Low CD4 and CD16	:::	Imiquimod UV protection	HPV vaccination	Alive
***P48***	***STK4***		F	3	16	Recurrent episodes of respiratory infections	Affected brother	Low CD4 Low CD19	**:::**	No	—	Alive
***P52***	***EPG5***	STXBP2	M	5	9	Repeated infections,Sarcoidosis like, lymphoproliferation, Cardiomyopathy	Cytopenia Eosinopenia Mother with splenomegalia/thrombopenia	Low CD4 and CD16	**SmB-TrNorm**	Corticosteroids	—	Alive
***P56***	***LRBA***	ITGAM/ PSTPIP1	F	1	4	Recurrent infections, IBD-like, lymphoproliferation, autoimmune cytopenia	Mother with hyperthyroidism	Neutropenia low CD 16	:::	Mycophenolate mofetil	:::_	Alive
***P58***	***RNASEH2B***	C7	M	14	14	Lymphoproliferatio, autoimmune cytopenias, repeated infections	Uveitis	Normal CD3, CD4,CD8CD19 CD16	**SmB-CD21low**	No	—	Alive
***P59***	***STK4***		F	3	8	Episodes of diarrhea and repeated respiratory infections, warts, pityriasis versicolor-like skin lesions	Skin abscesses	Low CD3, CD4,CD8CD19 CD16 Profound neutropenia	:::	Imiquimod UV protection	HPV vaccination	Alive
***P20***	***TNRFSF13B VUS (TACI)***	***CR2 (CD21 deficiency)***	F	14	15	Recurrent infections, IBD-like, lymphoproliferation, autoimmune cytopenia	Shingles	CD3, CD4,CD8CD19 CD16 Normal	**SmB-CD21low**	Azathioprine	No	Alive
***P64***	***LRBA***		F	2	4	Respiratory infections, lymphoproliferation, Granulomatosis, IBD-like	:::_	CD3, CD4,CD8CD19 normal Low CD16	:::	Corticosteroids	Infliximab	Alive
***P35***	***TNFRSF13B***		M	51	55	Repeated infections,Lymphoproliferation	Respiratory failure	CD3, CD4,CD8CD19 CD16 Normal	**SmB-TrHi**	Corticosteroids	:::	Alive
***P37***	***No gene*** ***found***		F	34	38	Repeated infections, autoimmune cytopenias		CD3, CD4,CD8CD19 CD16 Normal	:::	Corticosteroids	:::	Alive
***P43***	***No gene*** ***found***		F	29	30	Recurrent infections, granulomatosis,lymphoproliferation, autoimmune cytopenia	Arthritis	CD3, CD4,CD8CD19 CD16 Normal	**SmB-CD21low**	Corticosteroids	:::	Alive
***P51***	***No gene*** ***found***		M	1	4	Repeated deep-seated abscesses	Bullous dermatosis	CD3, CD4,CD8CD19 CD16 Normal	::_	Corticosteroids	::_	Alive
***P61***	***No gene*** ***found***		M	2	12	Recurrent infections, granulomatosis,lymphoproliferation		CD3, CD4,CD8CD19 normal Low CD16	::_	Corticosteroids	::_	Alive
***P45***	***No gene*** ***found***		F	1	9	Recurrent infections, IBD-like		CD3, CD4,CD8CD19 normal Low CD16	SmB-TrNorm	Corticosteroids	::_	Alive
***P31***	***No gene*** ***found***	DNM3	F	20	30	Evans+ splenomegaly	Arthritis	CD3, CD4,CD8CD19 CD16 Normal	**SmB-CD21low**	Corticosteroids+ Azathiorpine	No	Alive

F, female; M, male; CD, IBD, inflammatory bowel disease; cluster of differentiation, SmB, switched memory B lymphocytes; Trhi Transitional high.

Family segregation study was done for *STK4* gene in two siblings (P47, P48).

No functional studies were performed to evaluate the pathogenicity of identified variants.

### Subgroup analysis based on consanguinity

A total of 61 CVID patients were analyzed, of whom 33 (54.1%) were born from consanguineous unions. Subgroup analysis revealed several clinically and statistically meaningful differences. Patients from consanguineous families presented with an earlier mean age at disease onset compared to non-consanguineous individuals (16.2 ± 17.1 years *vs*. 21.3 ± 17.3 years; p < 0.001), and experienced a shorter diagnostic delay (5.8 ± 4.6 years *vs*. 7.9 ± 11.2 years; p = 0.12). Complications were more frequently observed in the consanguineous group, including a significantly higher rate of severe infections (75% *vs*. 25%; p = 0.03) and a trend toward increased bronchiectasis (59.2% *vs*. 40.8%; p = 0.06). Interestingly, immune dysregulation manifestations were less prevalent among consanguineous patients, with lower rates of autoimmunity (33.3% *vs*. 66.7%; p = 0.05), lymphoproliferation (40% *vs*. 60%; p = 0.44), and granulomatous disease (18.2% *vs*. 81.8%; p = 0.04). A disease-causing genetic variant was identified more frequently in consanguineous individuals (60% *vs*. 40%; p = 0.06), reinforcing the suspected higher burden of monogenic inborn errors of immunity in this subgroup. There was no significant difference in the use of intravenous immunoglobulin therapy between the two groups (85.7% *vs*. 87.9%; p = 0.80). Finally, the use of immunomodulatory or immunosuppressive treatments was more common in consanguineous patients (25% *vs*. 12.1%), although the difference did not reach statistical significance (p = 0.19).

## Discussion

Our study provides the first comprehensive and integrative overview of the clinical, immunological, and genetic spectrum of CVID patients in Morocco, a middle/low-income North African country. Our findings add to the growing body of literature on CVID in non-Western populations and highlight both similarities and specific challenges in diagnosis and management.

### Epidemiology and diagnostic challenges

CVID constituted 5.7% of all IEIs in the Moroccan registry reported in 2014 (10) and currently represents 8.2% of all IEIs in the Moroccan registry (742 cases) compiled by December 2024, suggesting enhanced disease recognition and improved diagnostic capabilities in this part of the world. Our study is in line with the prevalence registered in the IEI prevalence study in the MENA region, where CVID accounted for 11,3% (n=1935) (17). Potential reasons behind the low CVID account in Morocco could be underdiagnosis, registry bias and consanguinity-driven recessive IEIs in the MENA region (17).

The mean age at diagnosis was 25.9 (SD 18.7) years, which is older than previous reports from our country (29, 30), reflecting a better recognition of CVID by practitioners caring for adult patients. However, the mean diagnostic delay is significant, 6.91 (SD 8.82) years, consistent with reported literature (17, 31, 32).

In our cohort, the consanguinity rate was notably high at 54.1%, which is significantly greater than the average of 15-25% found in the general Moroccan population (33, 34). Our findings indicate that consanguinity may be linked to earlier disease onset, more severe infections, and a higher rate of genetic diagnoses in patients with CVID. However, these results should be interpreted with caution due to several limitations. The small sample size, the retrospective nature, restricted access to genetic testing, and potential underreporting of immune dysregulation features may have impacted the subgroup comparisons. Additionally, genetic analyses were only conducted in a subset of patients, which might underestimate the true prevalence of monogenic diseases. To confirm these trends and improve tailored management strategies for consanguineous populations, larger prospective studies with comprehensive genomic screening are necessary. Nevertheless, it is essential to systematically investigate parental consanguinity in patients with CVID, as it is often linked to an increased risk of developing severe clinical complications (35).

### Infectious and non-infectious complications

Pulmonary infections were the predominant infectious complication (88.5%), consistent with global data showing respiratory tract infections as the most common clinical manifestation of CVID (36, 37). Gastrointestinal infections, notably those caused by giardia enteritis, norovirus, and enterovirus, were also prevalent (63.9%), in line with previous studies indicating that up to 60% of CVID patients experience gastrointestinal infections (38). The high prevalence of Helicobacter pylori-induced gastritis and tuberculosis (11.5%) further underscores the regional burden of infectious diseases (39, 40).

Non-infectious complications were observed in 49.2% of the total cohort and were significantly more frequent in patients with a confirmed genetic defect (79%). This highlights the potential role of underlying monogenic etiologies in driving more severe phenotypes. Additionally, prolonged diagnostic delay and limited access to specialized care in our region may contribute to the higher burden of complications. Although data on atypical pathogens and IgG trough levels were not available for all patients, these factors may also have played a role and should be further explored in future studies.

Lymphoproliferation and splenomegaly being the most frequent (50.8%). These findings corroborate prior studies reporting lymphoproliferative disorders in nearly half of CVID patients (6). The prevalence of autoimmune cytopenias (39.3%), granulomatous disease (18%), and systemic autoimmune diseases (lupus, rheumatoid arthritis) aligns with previous research emphasizing the autoimmune predisposition in CVID (2). It underlies the paradoxical combination between immune deficiency and immune dysregulation in CVID and the difficulty of patients’ management to navigate all this combined complexity.

Bronchiectasis (44.3%) was the most frequent pulmonary complication, consistent with rates reported in European cohorts (41). It may be caused by repeated infections combined with local pulmonary immune dysregulation (41, 42).

The absence of histologically confirmed GLILD (granulomatous-lymphocytic interstitial lung disease) in our patients is notable, given its reported prevalence of 10-20% in Western cohorts (42, 43), and can be explained by the limited screening for chronic lung disease in our settings, especially in paucisymptomatic patients, and also by the lack of consensus on diagnostic criteria for CVID interstitial lung disease (43). This highlights the need for systematic and active screening for chronic lung disease in CVID patients, as it is associated with increased morbidity and mortality. Histologically confirmed enteropathy mimicking celiac disease (14.8%) and IBD-like disease (13.1%) reinforce the association between CVID and gastrointestinal autoimmunity and inflammation (38). Intestinal biopsies often show an increase in the number of intraepithelial lymphocytes, giving an appearance similar to lymphocytic colitis, but this intestinal inflammation in CVID is distinguished from classic IBD by the absence of plasma cells in the chorion (44).In celiac disease, the histology is also similar, except for the absence of plasma cells in the chorion in CVID, but with the constant presence of CD3+ CD8+ intraepithelial T lymphocytes (45). CVID patients have an increased risk of gastric cancer and digestive lymphoma (46). These findings further emphasize the need for early gastroenterological evaluation in CVID patients presenting with chronic diarrhea and weight loss.

### Immunological and genetic landscape

The immunoglobulin profile and lymphocyte subpopulation analysis in our cohort demonstrated significant heterogeneity, as reported in the literature (34). IgG hypogammaglobulinemia was the most consistent feature, with variable reductions in IgA and IgM. Decreased memory switched B cells was also the hallmark of immune dysregulation in our cohort (52,8% of patients with <2% switched memory B cells had immune dysregulation disorders), that was also noted in the Euroclass trial by Wher et al. (20).

While most cases of CVID are sporadic, it is estimated that 5-25% of cases run in families with transmission, most often autosomal dominant (3). It has been 22 years since the first genetic etiology of CVID was described, reporting homozygous mutations of *ICOS* of autosomal recessive transmission in patients from the same family (47). Since then, a growing number of genetic diseases responsible for CVID have been described, affecting up to 10% or even 20% of patients, particularly those with non-infectious manifestations (8, 48, 49). However, in consanguineous cohorts such as ours, monogenic disorders can account for approximately 70% of CVID patients (50). With this increasing number of genes described, it has become clear that CVID is an umbrella disease and that many of these genetic defects cause distinct disease entities, which we currently refer to as CVID-like diseases (49).

In our study, genetic testing using a targeted panel in 25 patients (41%) revealed 19 putative pathogenic variants in 13 genes, with novel variants in 73.7% of cases. The identification of mutations in *LRBA, CTLA4, PIK3CD, and NFKB1* is in line with emerging literature demonstrating the genetic heterogeneity of CVID (9–11). Notably, *STK4* deficiency was identified in three patients, a gene for combined immunodeficiencies; this type of entity is increasingly recognized in CVID-like phenotypes with combined immunodeficiency features (51, 52). While 52.6% of patients demonstrated a high phenotype-genotype correlation, the absence of functional validation remains a limitation.

Genetic testing in patients with CVID can potentially guide therapy, as was the case in our study, such as the use of abatacept, infliximab, and rituximab in the treatment of autoimmune manifestations of CVID in patients with *CTLA-4* and *LRBA* mutations. mTOR inhibitors can also be considered, especially in patients with *PI3K* signaling defects (7).

### Therapeutic management and outcomes

Immunoglobulin replacement therapy (IgRT) was administered exclusively through intravenous infusion to 86.9% of patients, leading to a reduction in the incidence of recurrent and severe infections as well as subsequent hospitalizations. However, due to the retrospective nature of our study, these results should be interpreted with caution.

The use of prophylactic antibiotics in 75.4% of cases is in line with international recommendations for infection prevention in CVID patients (41, 53), as some patients had persistent infections even after immunoglobulin replacement therapy.

The use of corticosteroids (49.2%) and immunosuppressive agents (16.4%) reflects the need for immune modulation in CVID-associated autoimmune and inflammatory complications. Targeted therapies, such as rituximab, abatacept, and infliximab, were used in 8.2% of cases, highlighting the evolving role of biologics in CVID management (54).

Mortality in our cohort was 4.9%, mainly due to infectious complications and dysimmune disorders. This rate is lower than in other large CVID studies (55, 56), which may be due to the short duration of the study and the small number of patients. Mortality is increased in CVID, 10 times higher than in the general population, as reported in some studies (55), highlighting the importance of early diagnosis and individualized therapeutic strategies.

### Strengths and limitations

Our study presents several strengths. It is the first comprehensive analysis of CVID in Morocco, contributing valuable epidemiological, clinical, and genetic data from a non-Western setting. The inclusion of genetic analysis provides novel insights into the molecular basis of CVID in this population. Additionally, our study highlights specific regional challenges, including diagnostic delays and a high burden of infectious diseases, thereby informing future healthcare strategies.

Several limitations of this study should be acknowledged. First, the relatively small sample size may restrict the generalizability of our findings to broader populations. Genetic testing was performed in only 41% of patients, and functional validation of novel variants could not be conducted, limiting our ability to confirm the pathogenicity of some genetic alterations. Moreover, due to the retrospective design, potential information bias may have affected the accuracy of recorded clinical complications and therapeutic outcomes. Another important limitation concerns the diagnostic workup: full immunophenotyping was not available for all patients due to technical constraints. We followed ESID and MENA definitions, but our decisions were also guided by expert consensus within our national immunology network, relying on clinical presentation, Ig levels, and exclusion of secondary causes. Nevertheless, the absence of full immunophenotyping and standardized functional immune evaluation in all patients reflects the technical and resource constraints of our setting. Future prospective studies with larger cohorts, comprehensive immunological assessment, and long-term follow-up are warranted to better characterize the phenotypic and genotypic diversity of CVID in North African populations.

## Conclusion

Our study underscores the clinical and immunological heterogeneity of CVID in a North African population, marked by a high burden of infectious and autoimmune complications and significant diagnostic delays. The substantial proportion of patients born to consanguineous unions and the high rate of monogenic diagnoses highlight the critical role of genetic factors in this setting. These findings reinforce the value of early genetic testing in consanguineous populations to enhance diagnostic accuracy, enable personalized management, and support genetic counseling. Incorporating genomic tools into national diagnostic protocols could significantly improve outcomes and reduce healthcare disparities in similar low- and middle-income contexts. Future efforts should focus on expanding access to genetic sequencing, functionally validating novel variants, and developing region-specific care strategies to optimize long-term outcomes for patients with CVID.

## Data Availability

The original contributions presented in the study are included in the article/**Supplementary Material**. Further inquiries can be directed to the corresponding author.
