# Supplementary material for: Clinical, immunological, and genetic landscape of common variable immunodeficiency in Morocco: a nationwide multicenter study
Source: Front Immunol. 2025 Jul 9;16:1602820. doi: 10.3389/fimmu.2025.1602820 (PMC12283719; doi:10.3389/fimmu.2025.1602820)
Supplement: Supplementary file 1 [file DataSheet1.pdf]

Details about the genetic testing panel (gene list, sequencing coverage, depth):

GENE	TRANSCRIPT
ABCB7	NM_004299.4
ABCG5	NM_022436.2
ABCG8	NM_022437.2
ACAN*	NM_013227.3
ACD	NM_001082486.1
ACP5	NM_001111035.2
ACTB	NM_001101.3
ACTN1	NM_001130004.1
ADA	NM_000022.2
ADA2	NM_001282225.1
ADAM17	NM_003183.5
ADAMTS13	NM_139025.4
ADAR	NM_001111.4
ADGRE2*	NM_013447.3
AICDA	NM_020661.2
AIRE	NM_000383.3
AK2*	NM_001625.3
AK7	NM_152327.3
ALAS2	NM_000032.4
ALG6	NM_013339.3
ALPK1*	NM_001102406.1
ANGPT1	NM_001146.4
ANKRD26*	NM_014915.2
ANKZF1	NM_018089.2
ANO6*	NM_001025356.2
AP3B1	NM_003664.4
AP3D1	NM_001261826.1
ARHGEF1	NM_199002.1
ARMC4*	NM_018076.2
ARPC1B	NM_005720.3
ASAH1	NM_177924.3
ATM*	NM_000051.3
ATP6AP1	NM_001183.5
ATR*	NM_001184.3

GENE	TRANSCRIPT
B2M	NM_004048.2
BACH2	NM_021813.3
BCL10	NM_003921.4
BCL11B	NM_138576.3
BLM	NM_000057.3
BLNK	NM_013314.3
BLOC1S3	NM_212550.4
BLOC1S6	NM_012388.3
BRCA1	NM_007294.3
BRCA2	NM_000059.3
BRIP1	NM_032043.2
BTK	NM_000061.2
C11orf70	NM_032930.2
C15orf41	NM_001130010.2
C17orf62	NM_001033046.3
C1QA	NM_015991.2
C1QB	NM_000491.3
C1QC	NM_172369.3
C1S	NM_201442.2
C2	NM_000063.5
C3	NM_000064.3
C5	NM_001735.2
C6	NM_000065.3
C7	NM_000587.2
C8A	NM_000562.2
C8B	NM_000066.3
C9	NM_001737.4
CARD11	NM_032415.5
CARD14	NM_024110.4
CARD8	NM_014959.3
CARD9	NM_052813.4
CARMIL2	NM_001013838.1
CASP10	NM_032977.3
CASP8	NM_001228.4

GENE	TRANSCRIPT
CBL	NM_005188.3
CCBE1	NM_133459.3
CCDC103	NM_213607.2
CCDC114	NM_144577.3
CCDC151	NM_145045.4
CCDC39	NM_181426.1
CCDC40	NM_017950.3
CCDC65	NM_033124.4
CCNO	NM_021147.4
CD19	NM_001770.5
CD247	NM_198053.2
CD27	NM_001242.4
CD3D	NM_000732.4
CD3E	NM_000733.3
CD3G	NM_000073.2
CD40	NM_001250.5
CD40LG	NM_000074.2
CD46	NM_002389.4
CD55	NM_000574.4
CD59	NM_203330.2
CD79A	NM_001783.3
CD79B	NM_000626.3
CD81	NM_004356.3
CD8A	NM_001768.6
CDAN1	NM_138477.2
CDC42	NM_001791.3
CDCA7	NM_031942.4
CEBPE	NM_001805.3
CEP164	NM_014956.4
CFAP298	NM_021254.2
CFB	NM_001710.5
CFD	NM_001928.3
CFH*	NM_000186.3
CFI	NM_000204.4

GENE	TRANSCRIPT
CFP	NM_002621.2
CFTR*	NM_000492.3
CHD7	NM_017780.3
CHEK2	NM_007194.3
CIB1	NM_001277764.1
CIITA	NM_000246.3
CLCN7	NM_001287.5
CLPB	NM_030813.5
COL7A1	NM_000094.3
COPA	NM_004371.3
CORO1A*	NM_007074.3
CR2	NM_001006658.2
CSF2RA*	NM_006140.4
CSF2RB	NM_000395.2
CSF3R	NM_000760.3
CTC1	NM_025099.5
CTLA4	NM_005214.4
CTPS1	NM_001905.3
CTSC	NM_001814.5
CXCR2	NM_001557.3
CXCR4	NM_003467.2
CYBA	NM_000101.3
CYBB	NM_000397.3
CYCS	NM_018947.5
CYP27A1	NM_000784.3
DBR1	NM_016216.3
DCLRE1C	NM_001033855.2
DDX41	NM_016222.3
DDX58	NM_014314.3
DEF6	NM_022047.3
DGAT1	NM_012079.5
DGKE	NM_003647.2
DIAPH1	NM_005219.4
DKC1	NM_001363.4
DNAAF1	NM_178452.4
DNAAF2	NM_018139.2
DNAAF3	NM_001256714.1
DNAAF4	NM_130810.3
DNAAF5	NM_017802.3

GENE	TRANSCRIPT
DNAH1	NM_015512.4
DNAH11	NM_001277115.1
DNAH5	NM_001369.2
DNAH8	NM_001206927.1
DNAH9	NM_001372.3
DNAI1	NM_012144.3;NM_001281428.1
DNAI2	NM_023036.4
DNAJB13	NM_153614.3
DNAJC21	NM_001012339.2
DNAL1	NM_031427.3
DNASE1L3	NM_004944.3
DNASE2	NM_001375.2
DNMT3B	NM_006892.3
DOCK2	NM_004946.2
DOCK8	NM_203447.3
DRC1	NM_145038.3
DSG1	NM_001942.3
DTNBP1	NM_032122.4
DUOX2*	NM_014080.4
EFL1*	NM_024580.5
EIF2AK3	NM_004836.6
ELANE	NM_001972.2
EPCAM*	NM_002354.2
EPG5	NM_020964.2
ERBIN	NM_001253697.1
ERCC2	NM_000400.3
ERCC3	NM_000122.1
ERCC4	NM_005236.2
ERCC6L2	NM_020207.4
ETV6	NM_001987.4
EXTL3	NM_001440.3
FADD	NM_003824.3
FANCA	NM_000135.2
FANCB	NM_001018113.1
FANCC	NM_000136.2
FANCD2*	NM_033084.3
FANCE	NM_021922.2
FANCF	NM_022725.3
FANCG	NM_004629.1

GENE	TRANSCRIPT
FANCI	NM_001113378.1
FANCL*	NM_018062.3
FANCM	NM_020937.2
FAS	NM_000043.5
FASLG	NM_000639.2
FAT4	NM_024582.4
FCHO1	NM_001161357.1
FERMT1	NM_017671.4
FERMT3	NM_031471.5
FLI1	NM_002017.4
FNIP1	NM_133372.2
FOXI3	NM_001135649.2
FOXN1	NM_003593.2
FOXP3	NM_014009.3
FPR1	NM_002029.3
G6PC	NM_000151.3
G6PC3	NM_138387.3
G6PD	NM_001042351.2
GAS8	NM_001481.2
GATA1	NM_002049.3
GATA2	NM_032638.4
GFI1*	NM_005263.3
GINS1	NM_021067.4
GLRX5	NM_016417.2
GP1BA*	NM_000173.6
GP6	NM_001083899.2
GP9	NM_000174.4
GTF2E2	NM_002095.4
GTF2H5	NM_207118.2
GUCY2C	NM_004963.3
HAX1	NM_006118.3
HELLS	NM_018063.4
HMOX1	NM_002133.2
HPS1	NM_000195.4
HPS3	NM_032383.4
HPS4	NM_022081.5
HPS5	NM_181507.1
HPS6	NM_024747.5
HTRA2	NM_013247.4

GENE	TRANSCRIPT
HYOU1	NM_006389.4
ICOS	NM_012092.3
ICOSLG	NM_015259.5
IFIH1	NM_022168.3
IFNAR1	NM_000629.2
IFNAR2	NM_207585.2
IFNGR1	NM_000416.2
IFNGR2*	NM_005534.3
IGLL1	NM_020070.3
IKBKB	NM_001556.2
IKZF1	NM_006060.6
IL10	NM_000572.2
IL10RA	NM_001558.3
IL10RB	NM_000628.4
IL12B	NM_002187.2
IL12RB1	NM_005535.2
IL12RB2	NM_001559.2
IL17F	NM_052872.3
IL17RA	NM_014339.6
IL17RC	NM_153461.3
IL18BP	NM_173042.2
IL1RN	NM_173841.2
IL21	NM_021803.3
IL21R	NM_021798.3
IL23R	NM_144701.2
IL2RA	NM_000417.2
IL2RB	NM_000878.3
IL2RG	NM_000206.2
IL36RN	NM_012275.2
IL6R	NM_000565.3
IL6ST	NM_002184.3
IL7R	NM_002185.3
IRAK4	NM_016123.3
IRF2BP2	NM_182972.2
IRF4	NM_002460.3
IRF7	NM_004031.2
IRF8	NM_002163.2
IRF9	NM_006084.4
ISG15	NM_005101.3

GENE	TRANSCRIPT
ITCH	NM_031483.6
ITGA2B	NM_000419.3
ITGAM	NM_000632.3
ITGB2	NM_000211.4
ITGB3	NM_000212.2
ITK	NM_005546.3
JAGN1	NM_032492.3
JAK1	NM_002227.3
JAK2	NM_004972.3
JAK3	NM_000215.3
KAT6A	NM_006766.4
KDM1A	NM_001009999.2
KDM6A*	NM_021140.3
KIF23	NM_138555.3
KIT	NM_000222.2
KLF1	NM_006563.4
KLHDC8B	NM_173546.2
KMT2A	NM_001197104.1
KMT2D	NM_003482.3
LAMTOR2	NM_014017.3
LARS2	NM_015340.3
LAT	NM_001014987.1
LCK	NM_001042771.2
LCT	NM_002299.3
LIG1	NM_000234.2
LIG4	NM_002312.3
LIPA	NM_000235.3
LPIN2	NM_014646.2
LRBA	NM_006726.4
LRRC56	NM_198075.3
LRRC6	NM_012472.4
LRRC8A	NM_019594.3
LYN	NM_002350.3
LYST	NM_000081.3
MAD2L2	NM_001127325.1
MAGT1	NM_032121.5
MALT1	NM_006785.3
MAP3K14	NM_003954.4
MBD4	NM_003925.2

GENE	TRANSCRIPT
MCIDAS	NM_001190787.1
MCM4	NM_005914.3
MECOM	NM_001105078.3
MEFV	NM_000243.2
MKL1	NM_020831.4
MLH1*	NM_000249.3
MOGS	NM_006302.2
MPL	NM_005373.2
MPLKIP	NM_138701.3
MS4A1	NM_152866.2
MSH2*	NM_000251.2
MSH6*	NM_000179.2
MSN	NM_002444.2
MTHFD1	NM_005956.3
MVK	NM_000431.3
MYD88	NM_002468.4
MYH9	NM_002473.5
MYO5B	NM_001080467.2
MYSM1	NM_001085487.2
NAF1	NM_138386.2
NBAS	NM_015909.3
NBN	NM_002485.4
NCF2	NM_000433.3
NCF4	NM_013416.3
NCKAP1L	NM_005337.4
NCSTN	NM_015331.2
NDUFB11*	NM_019056.6
NEUROG3	NM_020999.3
NF1*	NM_000267.3
NFAT5	NM_138714.3
NFE2L2	NM_006164.4
NFKB1	NM_003998.3
NFKB2	NM_001077494.3
NFKBIA	NM_020529.2
NHEJ1	NM_024782.2
NHP2	NM_017838.3
NLRC4	NM_021209.4
NLRP1	NM_033004.3
NLRP12	NM_144687.3

GENE	TRANSCRIPT
SEMA3E	NM_012431.2
SERPING1	NM_000062.2
SGPL1	NM_003901.3
SH2D1A	NM_002351.4
SH3BP2	NM_003023.4
SH3KBP1	NM_031892.2
SI*	NM_001041.3
SIAE	NM_170601.4
SKIV2L	NM_006929.4
SLC10A2	NM_000452.2
SLC19A2	NM_006996.2
SLC25A38	NM_017875.2
SLC26A3	NM_000111.2
SLC29A3	NM_018344.5
SLC35C1	NM_018389.4
SLC37A4	NM_001164277.1
SLC39A7	NM_001077516.1
SLC46A1	NM_080669.5
SLC51B	NM_178859.3
SLC5A1	NM_000343.3
SLC7A7	NM_001126106.2
SLC9A3*	NM_004174.3
SLX4	NM_032444.2
SMARCAL1	NM_014140.3
SMARCD2	NM_001098426.1
SNX10	NM_001199835.1
SP110	NM_004509.3
SPAG1	NM_172218.2
SPINK5	NM_006846.3
SPINT2	NM_021102.3
SPPL2A	NM_032802.3
SRP54	NM_003136.3
SRP72	NM_006947.3
STAT1	NM_007315.3
STAT2	NM_005419.3
STAT3	NM_139276.2
STAT4	NM_003151.3
STAT5B*	NM_012448.3
STIM1	NM_003156.3

GENE	TRANSCRIPT
STK4	NM_006282.3
STN1	NM_024928.4
STX11	NM_003764.3
STX3	NM_004177.4
STXBP2	NM_006949.3
TAOK2	NM_016151.3
TAP1	NM_000593.5
TAP2	NM_000544.3
TAPBP	NM_003190.4
TAZ	NM_000116.4
TBX1	NM_080647.1
TBXA2R	NM_001060.5
TCF3	NM_003200.4;NM_001136139.3
TCIRG1	NM_006019.3
TCN2	NM_000355.3
TERC	NR_001566.1
TERF2IP	NM_018975.3
TERT	NM_198253.2
TET2	NM_001127208.2
TFRC	NM_003234.3
TGFB1	NM_000660.5
TGFBR1	NM_004612.2
TGFBR2	NM_003242.5
THBD	NM_000361.2
THPO	NM_000460.3
TICAM1	NM_182919.3
TIMM50	NM_001001563.3
TINF2	NM_001099274.1
TLR3	NM_003265.2
TLR7	NM_016562.3
TMC6	NM_007267.7
TMC8	NM_152468.4
TMEM173	NM_198282.3
TMPRSS15	NM_002772.2
TNFAIP3	NM_006290.3
TNFRSF11A	NM_003839.3
TNFRSF13B	NM_012452.2
TNFRSF13C	NM_052945.3
TNFRSF1A	NM_001065.3

GENE	TRANSCRIPT
TNFRSF4	NM_003327.3
TNFRSF6B	NM_003823.3
TNFRSF9	NM_001561.5
TNFSF11	NM_003701.3
TNFSF12	NM_003809.2
TONSL	NM_013432.4
TOP2B*	NM_001068.3
TP53	NM_000546.5
TP63	NM_003722.4
TPP2	NM_003291.2
TRAF3	NM_003300.3
TRAF3IP2	NM_147686.3
TREX1	NM_033629.4
TRNT1	NM_182916.2
TSR2	NM_058163.2
TTC37	NM_014639.3
TTC7A	NM_020458.3
TUBB1	NM_030773.3
TYK2	NM_003331.4
UBE2T	NM_014176.3
UNC13D	NM_199242.2
UNC45A	NM_018671.4
UNC93B1*	NM_030930.3
UNG	NM_080911.2
USB1	NM_024598.3
VAV1	NM_005428.3
VIPAS39	NM_022067.3
VPS13B	NM_017890.4
VPS33B	NM_018668.4
VPS45	NM_007259.4
WAS	NM_000377.2
WDR1	NM_017491.3
WIPF1	NM_001077269.1
WNT2B	NM_024494.2
WRAP53	NM_018081.2
XIAP	NM_001167.3
XRCC2	NM_005431.1
YARS2	NM_001040436.2
ZAP70	NM_001079.3

GENE	TRANSCRIPT
ZBTB24	NM_014797.2
ZCCHC8	NM_017612.4
ZMYND10	NM_015896.2
ZNF341	NM_032819.4

Samples are enriched for targeted regions using a hybridization-based protocol, and sequenced using Illumina technology

Depth and sequence coverage :

All targeted regions were sequenced with ≥50x depth. Reads were aligned to a reference sequence (GRCh37), and sequence changes were identified and interpreted in the context of a single clinically relevant transcript, indicated in the Genes Analyzed table.

The assay achieved>99% analytical sensitivity and specificity for single nucleotide variants, insertions and deletions <15bp in length, and exon-level deletions and duplications. The methods used also detected insertions and deletions larger than 15bp but smaller than a full exon but sensitivity for these may be marginally reduced.
