# Supplementary material for: Clinical, immunological, and genetic landscape of common variable immunodeficiency in Morocco: a nationwide multicenter study
Source: Front Immunol. 2025 Jul 9;16:1602820. doi: 10.3389/fimmu.2025.1602820 (PMC12283719; doi:10.3389/fimmu.2025.1602820)
Supplement: Supplementary file 2 [file DataSheet2.pdf]

Supplementary Table 2 « Matrix of the clinical and genetic features of Moroccan patients with CVID »

Patient	Sex	Consanguinity	Ageofonset	Bronchiostasis	Lymphoproliferation	Autoimmunity	Switched Memory B cells	Genetic diagnosi	Diagnosis delay
**3**	Femal	Yes	8	Yes	No	Yes	B absent	*LRBA*	7
**1**	Femal	Yes	4	Yes	Yes	Yes	<2%	*VAV1*	12
**14**	Mal	Yes	21	No	No	Yes	<2%	*CTPS1*	8
**16**	Mal	Yes	5	Yes	No	No	B absent	*PIK3CD*	4
**20**	Femal	Yes	14	Yes	No	No	<2%	*TNRFSF13B*	1
**27**	Mal	No	4	Yes	No	No	<2%	*LRBA*	61
**28**	Mal	No	13	No	No	Yes	<2%	*TCF3*	4
**33**	Mal	No	13	Yes	Yes	Yes	B absent	*CTLA4*	7
**34**	Femal	No	20	No	No	Yes	<2%	*CD19*	13
**35**	Mal	Yes	51	Yes	No	No	<2%	*TNFRSF*[illegible]	4
**37**	Femal	Yes	34	Yes	No	Yes	N/A	No variant	4
**2**	Mal	No	23	Yes	No	No	>2%	N/A	1
**4**	Mal	No	36	Yes	No	No	>2%	N/A	24
**5**	Mal	No	17	Yes	Yes	Yes	<2%	N/A	13
**6**	Mal	No	15	Yes	No	No	<2%	N/A	5
**7**	Femal	No	18	Yes	No	No	N/A	N/A	8

**8**	Femal	Yes	12	Yes	No	No	N/A	N/A	3
**9**	Femal	No	18	No	Yes	Yes	<2%	N/A	1
**10**	Femal	No	29	No	No	Yes	<2%	N/A	1
**11**	Femal	No	45	No	Yes	Yes	<2%	N/A	1
**12**	Femal	Yes	1	No	No	No	<2%	N/A	14
**13**	Femal	Yes	9	No	No	No	<2%	N/A	2
**15**	Mal	No	3	No	Yes	Yes	<2%	N/A	4
**17**	Femal	No	27	No	Yes	Yes	N/A	N/A	3
**18**	Mal	No	27	No	Yes	Yes	N/A	N/A	7
**19**	Mal	No	8	No	No	Yes	<2%	N/A	1
**21**	Femal	Yes	55	Yes	Yes	Yes	N/A	N/A	2
**22**	Mal	No	56	No	Yes	Yes	N/A	N/A	2
**23**	Femal	No	12	No	No	No	N/A	N/A	23
**24**	Mal	Yes	2	Yes	Yes	No	N/A	N/A	9
**25**	Femal	Yes	5	Yes	Yes	No	N/A	N/A	8
**26**	Femal	Yes	19	Yes	No	No	<2%	N/A	7
**29**	Mal	Yes	46	Yes	Yes	Yes	<2%	N/A	10
**30**	Femal	No	61	No	No	Yes	<2%	N/A	5

**31**	Femal	No	29	No	No	Yes	<2%	No variant	1
**36**	Mal	No	66	Yes	No	Yes	<2%	N/A	12
**42**	Mal	Yes	38	Yes	Yes	Yes	<2%	N/A	16
**40**	Mal	No	10	No	No	No	<2%	*SH3KBP*	2
**44**	Mal	Yes	17	No	Yes	No	N/A	N/A	-4
**43**	Femal	No	29	Yes	Yes	No	<2%	No variant	15
**58**	Mal	No	14	No	Yes	Yes	<2%	*RNASEH*	0
**50**	Femal	No	26	No	Yes	Yes	<2%	N/A	7
**49**	Mal	No	2	No	Yes	No	N/A	N/A	3
**52**	Mal	No	5	No	Yes	Yes	N/A	*EPG5*	4
**51**	Mal	Yes	1	No	No	Yes	N/A	No varia	3
**53**	Mal	Yes	39	Yes	Yes	Yes	N/A	N/A	3
**57**	Mal	No	27	No	No	No	<2%	N/A	3
**59**	Femal	Yes	8	No	No	No	N/A	*STK4*	3
**64**	Femal	Yes	2	No	Yes	No	N/A	*LRBA*	2
**61**	Mal	Yes	2	No	Yes	No	N/A	No varia	10
**62**	Mal	No	2	Yes	Yes	No	<2%	N/A	3
**45**	Femal	Yes	1	Yes	No	No	<2%	No variant	8
**47**	Mal	Yes	10	No	No	No	N/A	*STK4*	2

**48**	Femal	Yes	3	No	No	No	N/A	*STK4*	13
**46**	Femal	No	40	No	No	Yes	>2%	N/A	4
**56**	Femal	Yes	1	No	No	Yes	N/A	*LRBA*	3
**60**	Mal	No	2	No	Yes	Yes	N/A	*NFKB1*	4
**41**	Mal	Yes	40	Yes	No	No	<2%	N/A	2
**65**	Femal	No	2	Yes	No	No	<2%	N/A	8
**66**	Mal	No	3	No	No	No	<2%	N/A	10
**67**	Mal	Yes	6	No	No	No	<2%	N/A	6
