# Supplementary material for: Clinical, immunological, and genetic landscape of common variable immunodeficiency in Morocco: a nationwide multicenter study
Source: Front Immunol. 2025 Jul 9;16:1602820. doi: 10.3389/fimmu.2025.1602820 (PMC12283719; doi:10.3389/fimmu.2025.1602820)
Supplement: Supplementary file 3 [file DataSheet3.pdf]

Supplementary table 3: Immunomodulatory treatments in Moroccan patients with CVID

Patient ID	Indication	Drug	Dose/ Schedule	Clinical Response	Adverse Events	Genetic diagnosis
**P7**	Autoimmune cytopenia	Rituximab	375 mg/m² weekly × 4	Complete response	None	N/A
**P14**	Burkitt lymphoma	Rituximab	375 mg/m² weekly × 5	Remission	N/A	*CTPS1*
**P36**	Marginal-zone lymphoma	Rituximab	375 mg/m² weekly × 6	Remission	N/A	N/A
**P3**	IBD-like enteropathy	Abatacept	10 mg/kg monthly	stopped for Partial improvement	Mild infections	*LRBA*
**P33**	Inflammatory enteropathy	Abatacept	10 mg/kg monthly	Partial improvement	N/A	*CTLA4*
**P3**	Arthritis + IBD-like enteropathy	Infliximab	5 mg/kg increased to 10mg/kg every 8 weeks	Moderate response	Transient rash	*LRBA*
**P64**	IBD-like enteropathy	Infliximab	5 mg/kg every 8 weeks	Moderate response	N/A	*LRBA*
**P33**	Psoriatic rheumatism	Methotrexate	15 mg/week	Moderate control	Mild liver enzyme [illegible]	*CTLA4*
**P36**	Rheumatoid arthritis	Methotrexate	15 mg/week	Good response	Alopecia	N/A
**P31**	Evans syndrome	Azathioprine	2.5mg/kg/day	Good response	None	No variant
**P20**	Autoimmune cytopenia + IBD-like enteropathy	Azathioprine	2.5mg/kg/day	N/A	N/A	*TNRFSF13B*
**P62**	Autoimmune cytopenia	Azathioprine	2.5mg/kg/day	N/A	N/A	N/A
**P3**	Autoimmune cytopenia	Mycophenolate mofetil	2g/day	Moderate response, stopped	Abdominal pain	*LRBA*
**P56**	Autoimmune cytopenia	Mycophenolate mofetil	2g/day	Remission	Mild liver enzyme elevation	*LRBA*
**P3**	Arthritis	Hydroxychloroquin	5mg/kg/day	Moderate response	None	*LRBA*
